# Assessment of the relationship between the level of patient knowledge on warfarin therapy and the quality of oral anticoagulation: A systematic review and meta-analysis

**DOI:** 10.1371/journal.pone.0289836

**Published:** 2023-08-10

**Authors:** Marcus Fernando da Silva Praxedes, José Luiz Padilha da Silva, Ana Júlia Alves da Cruz, Catiane Costa Viana, Hannah Cardoso Barbosa, Nathália Sernizon Guimarães, Maria Auxiliadora Parreiras Martins

**Affiliations:** 1 Centro de Ciências da Saúde, Universidade Federal do Recôncavo da Bahia, Santo Antônio de Jesus, Brazil; 2 Departamento de Estatística, Universidade Federal do Paraná, Curitiba, Brazil; 3 Faculdade de Farmácia, Universidade Federal de Minas Gerais, Belo Horizonte, Brazil; 4 Faculdade de Medicina da Universidade Federal de Minas Gerais, Belo Horizonte, Brazil; 5 Ciências Médicas de Minas Gerais, Belo Horizonte, Brazil; Federal University of Minas Gerais, BRAZIL

## Abstract

The present study aimed to investigate the relationship between the level of patient knowledge on warfarin therapy and the quality of oral anticoagulation. This is a systematic review and meta-analysis written on the basis of Preferred Reporting Items for Systematic Reviews and Meta-Analyses (PRISMA) guideline. Searches at MEDLINE, EMBASE, Scopus and LILACS electronic databases were carried out on February 13, 2023, using the descriptors "Patient Medication Knowledge", "Patient Education as Topic", "Health Education", "Patient Education" and Warfarin. The steps of selection, data extraction and quality analysis of articles were performed independently by two reviewers. The analysis was performed considering patient knowledge as a possible modifier of time in therapeutic range (TTR). The meta-analysis included studies that reported the correlation coefficient (Pearson or Spearman) between patient knowledge and TTR. A subgroup analysis was performed according to questionnaires employed to measure patient knowledge. Twelve studies were selected with an overall sample size of 7634 participants and mean age 58.2 (standard deviation (SD)±12,8) years. Eleven (92.0%) cross-sectional studies. The mean TTR was 57.8% (SD±11,3%) and the average level of knowledge was 60.4%. The meta-analysis indicated that patient level of knowledge on warfarin therapy was moderately associated with TTR (rs = 0.435; 95% confidence interval (CI) = 0.163–0.645; I^2^ = 96%). Subgroup analysis indicated association between knowledge level and TTR in studies employing the OAK test (rs = 0.617; 95% CI = 0.192–0.847; I^2^ = 97%) and the AKA (rs = 0.269; 95% CI = 0.002 to 0.501; I^2^ = 94%). However, the subgroup analysis presented no significant difference between them (p = 0.14). The meta-regression showed a non-significant negative effect of age on the correlation (estimate = -0.028, 95% CI = -0.073 to 0.016, p = 0.207). No publication bias was noted (p = 0.881). To our knowledge, this is the first systematic review and meta-analysis gathering evidence about the relationship between the level of patient knowledge on oral anticoagulation with warfarin and TTR. The implementation of structured and patient-centered educational interventions is essential to effectively increase the level of patient knowledge and, thus, to improve the quality and safety of warfarin therapy. Systematic review registration number: PROSPERO CRD42023398030.

## Introduction

Anticoagulation therapy is recommended for primary and secondary thromboprophylaxis, and can be performed with oral anticoagulants (OACs). Vitamin K inhibitors, such as warfarin, and direct OACs, including direct Factor Xa inhibitors and Factor IIa inhibitors, are currently available in clinical practice. Direct OACs have better dose response predictability and do not require constant laboratory monitoring. However, their safe use has not yet been defined for patients with mechanical heart valves, atrial fibrillation (AF) with rheumatic heart disease and mitral stenosis, renal failure, pregnancy, and breastfeeding. Warfarin, therefore, is still widely prescribed worldwide, being the OAC of choice in low- and middle-income countries [1–3].

Despite the benefits of warfarin in clinical practice, it presents narrow therapeutic index, great variability in dose-response, influence of genetic polymorphisms, and interactions with numerous drugs, foods, and alcohol [2,4,5]. Thus, warfarin is among the main drugs associated with adverse events, including accidental deaths.

The adequate management of warfarin therapy becomes very important to prevent patient harm. Treatment monitoring is performed by assessing the international normalized ratio (INR) obtained from the prothrombin time. Sequential INR values can be used to calculate the time in therapeutic range (TTR), a measurement of the quality of oral anticoagulation which correlates with the risk of bleeding and thromboembolism [6]. The method proposed by Rosendaal is the most widely used to determine TTR. It is based on a linear interpolation using at least two INR values with a range from 0% to 100% [7]. Values above 60% define an adequate control, associated with lower risk of warfarin-related adverse events [8].

Patients’ knowledge about oral anticoagulation plays a key role in the prevention of complications, since the risk of bleeding or thromboembolism increases with inappropriate warfarin use [1,9]. The definition of patients´ knowledge comprises the awareness of drug name, indication, dosing regimen, adverse effects or side effects, and special administration instructions [10].

In this sense, assessing the current level of patient knowledge is the first step in planning interventions to improve the quality of oral anticoagulation. Deficiencies in patients´ knowledge can be identified and minimized by offering educational interventions focused on the establishment of a continuous program to improve the quality of oral anticoagulation. Studies have pointed out that patients with a better level of knowledge about warfarin therapy present better quality of oral anticoagulation [9,11,12]. However, this association has not been confirmed by other authors [13,14], demonstrating the lack of consensus in the literature. Thus, the aim of this study was to investigate the relationship between the patient level of knowledge on warfarin therapy and the quality of oral anticoagulation.

## Materials and methods

This is a systematic review and meta-analysis written on the basis of Preferred Reporting Items for Systematic Reviews and Meta-Analyses (PRISMA) guideline [15] (S1 Checklist). This review protocol was registered with PROSPERO under the registration number CRD42023398030.

### Inclusion criteria

Observational studies assessing the association between the patient level of knowledge on warfarin therapy and the quality of oral anticoagulation measured by TTR, calculated by Rosendaal’s method [7], were included. There was no language or location restriction. Duplicate studies, narrative, integrative, overviews, rapid reviews, scoping reviews or systematic reviews, meta-analyses, case reports, case series, and experimental animal studies were excluded.

### Data sources and search strategy

A search in the electronic databases MEDLINE, EMBASE, Scopus and LILACS was performed using the descriptors "Patient Medication Knowledge", "Patient Education as Topic", "Health Education", "Patient Education" and Warfarin. The search strategy combined free and indexed terms developed primarily for searching articles in MEDLINE, applying Medical Subject Heading (MeSH) terms, and adapted to the other databases. Unpublished studies and gray literature were searched through MedNar, OpenGrey, Google Scholar, and ProQuest Dissertations and Theses. The databases were searched for additional studies with no time limit on publication. The search was conducted on February 13, 2023. A description of the search terms and strategies by database is provided in S1 Appendix.

### Study selection

The studies were selected in two steps according to the eligibility criteria using the PRISMA [15] flowchart. Initially, studies were selected by reading titles and abstracts. Then, textual analysis was performed by reading the articles in full. All steps were performed independently by two reviewers (MFSP, AJAC). After the comparison of results, discrepancies were discussed with a third reviewer (MAPM) to reach consensus, if necessary.

### Quality assessment

Two authors (MFSP, AJAC) independently assessed the risk of bias of selected studies and any differences in results were resolved by consensus. The methodological quality of cohort studies was evaluated as proposed by the Newcastle-Ottawa Scale [16], considering high quality (>8 stars), medium (6–7 stars), or low quality (<6 stars). Cross-sectional studies were evaluated with the Agency for Research and Health Quality (ARHQ) Methodology Checklist for Cross-Sectional/Prevalence Studies, which consists of 11 items with answers such as "yes", "no" or "uncertain". Studies will be rated as "low quality" (<3 answer "yes"), "moderate quality" (4–7 answers "yes") or "high quality" (>8 answers "yes") [17]. Contacting the authors of the articles was considered if the full article or additional data when needed.

### Data extraction

Data were extracted from independently selected studies by two reviewers (MFSP, JLPS), included population information, study methods, exposures and outcomes as follows: authors, year, country, study design, sample size, main indications, age, TTR, knowledge test, score for knowledge, knowledge group and main result. Any disagreements between the reviewers were resolved by consensus, or with a third reviewer (MAPM). The authors reviewed the available data and the impact of missing data was considered as a limitation of the study, when applicable.

### Data synthesis

The analysis was performed considering that patient level of knowledge on warfarin therapy is a possible modifier of TTR. The meta-analysis included studies that reported the correlation coefficient between patient level of knowledge and the TTR. For each study, the correlation coefficient and the sample used were extracted. In all studies, Spearman’s rank correlation coefficient (rs) was used to measure association. The mean TTR and the mean level of knowledge were calculated using arithmetic means, according to the available data.

A meta-analysis of correlations was performed to summarize the results. To account for the underlying heterogeneity among the population studies, the random effects models were used. Fisher’s z transformation of correlations was used, as suggested in the literature [18]. Meta-analysis results were back transformed, that is, presented as correlations in text and figures. The between-study variance was estimated via restricted maximum-likelihood method and the inverse variance weighting was used for pooling the correlations. A subgroup analysis was performed according to the questionnaires employed to measure patient knowledge. A meta-regression analysis with age as a continuous covariate was performed to measure the potential influence of age on the correlation.

All model estimates were shown with 95% confidence interval (CI), and Q-statistic I^2^ and tau-squared statistics were used to assess heterogeneity between studies. Forest plots for all meta-analyses were provided. Publication bias was investigated using the Begg-Mazumdar test. A p-value <0.05 was considered statistically significant. All analyses were performed in R version 4.2.3 (R Core Team, 2020) using the meta package.

## Results

**Fig 1:**
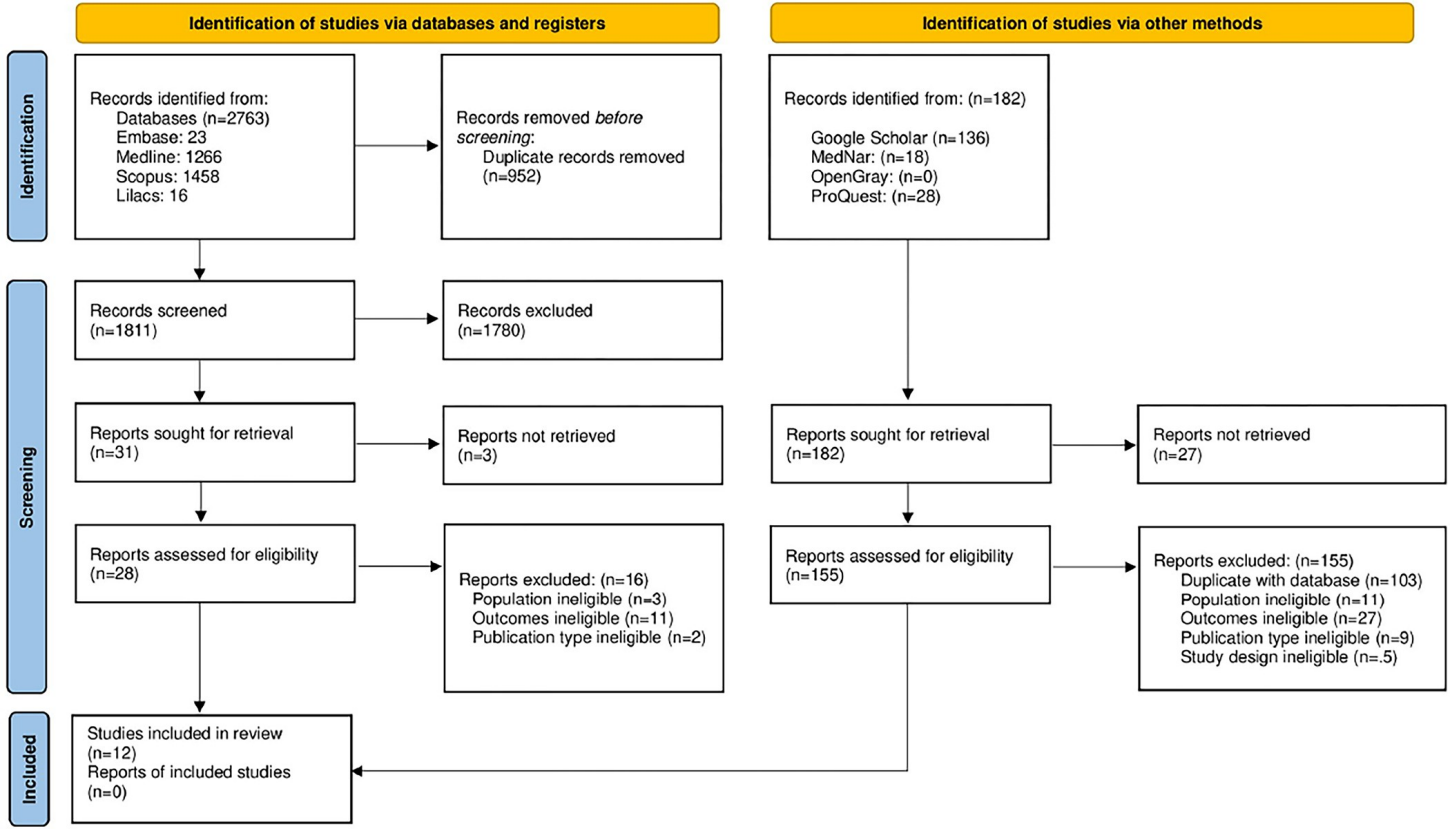
PRISMA 2020 flow diagram.

A total of 2763 records were retrieved after running the initial search. Overall, 12 studies [9,19–29] met the inclusion criteria of this systematic review, being selected for synthesis. A manual search was performed, with no identification of any study meeting the eligibility criteria. The flowchart of the process of selecting studies is depicted in Fig 1.

According to the data provided by the studies and presented in Table 1, five (41.7%) were developed in middle eastern countries, 11 (92.0%) were cross-sectional studies, with a total sample size of 7634 participants and mean age 58.2 (standard deviation (SD)±12.8) years. The arithmetic mean TTR was 57.8% (SD±11.3%). Six (50.0%) studies used the Anticoagulation Knowledge Assessment (AKA) instrument and found a mean knowledge level of 60.4%. According to the methodological quality assessment, all studies showed moderate quality.

**Table 1:** Characteristics of studies included in this systematic review and meta-analysis.

Authors/year	Country	Study design	Sample size (N)	Main indications	Age	TTR	Knowledge Test	Score for knowledge	Knowledge group	Main result	Methodological quality
Ahmed et al., 2021 [19]	Libya	Cross-Sectional	88	Mitral valve replacement	NA	63.8	OAK	54.5%	poor knowledge (<70%), n = 60 (68.2%) adequate knowledge (>70%), n = 28 (31.8%)	rs = 0.728, p<0.0001	Moderate quality
Baker et al., 2011 [20]	United States	Cross-Sectional	167	Atrial fibrillation	68.0	NA	AKA	78.1%	poor knowledge (<72.4%), n = 48 (25.9%) adequate knowledge (>72.4%), n = 137(74.1%)	rs = 0.015, p = 0.848	Moderate quality
Cao et al., 2020 [21]	China	Cross-Sectional	383	Heart valve replacement	50.3	60.3	AKA	62.3%	poor knowledge (<72.4%), n = 213 (55.6%) adequate knowledge (>72.4%), n = 170 (44.4%)	rs = 0.539, p<0.001	Moderate quality
Çelik et al., 2016 [22]	Turkey	Prospective Cohort	4987	Mechanical valve	60.7	49.5	Questionnaire not validated	55.0%	NA	p<0.001	Moderate quality
Feldeisen et al., 2023 [23]	United States	Cross-Sectional	481	NA	68.4	65.5	OAK	73.0%	poor knowledge (<70%), n = 293 (60.9%) adequate knowledge (≥70%), n = 188 (39.1%)	p = 0.020	Moderate quality
Li et al., 2018 [24]	China	Cross-Sectional	65	Atrial fibrillation	67.4	49.8	AKA	53.8%	poor knowledge (<72.4%), n = 59 (90.8%) adequate knowledge (>72.4%), n = 6 (9.2%)	rs = 0.356, p = 0.004	Moderate quality
Matalqah et al., 2013 [25]	Malaysia	Cross-Sectional	196	Atrial fibrillation	61.1	53.6	OAK	47.6%	low level of knowledge (<50%), 33.4±11.0 moderate level of knowledge (50–75%), 57.2±5.8 high level of knowledge (>75%), 80.4±7.2	rs = 0.192 p = 0.012	Moderate quality
Mayet, 2015 [26]	Saudi Arabia	Cross-Sectional	105	Deep vein thrombosis/ pulmonary embolism	55.4	NA	AKA	NA	poor knowledge (<75%), n = 79 (75.2%) adequate knowledge (≥75%), n = 26 (24.8%)	OR: 1.35(0.537–3.392) p = 0.522	Moderate quality
Praxedes et al., 2016 [9]	Brazil	Cross-Sectional	201	Atrial fibrillation/ Flutter	55.0	62.9	OAK	63.0%	low level of knowledge (<50%), n = 59 (29%) moderate level of knowledge (50–75%), n = 83 (42%) high level of knowledge (>75%), n = 76 (29%)	rs = 0.780; p = 0.001	Moderate quality
Shilbayeh et al., 2018 [27]	Saudi Arabia	Cross-Sectional	101	Deep vein thrombosis/ pulmonary embolism	55.1	52.1	AKA	46.1%	Satisfactory (≥50%), n = 49(48.5%) Unsatisfactory (<50%), n = 52 (51.5%)	rs = 0.101; p = 0.313	Moderate quality
Türker et al., 2021 [28]	Turkey	Cross-Sectional	240	Prosthetic heart valve	59.8	52.2	OAK	71.0%	low level of knowledge (<50%), n = 21±8.8 moderate level of knowledge (50–75%), n = 134±55.8 high level of knowledge (>75%), n = 85±35.4	p>0.05	Moderate quality
Zangenehfar et al., 2021 [29]	Islamic Republic of Iran	Cross-Sectional	620	Mitral valve replacement	52.4	NA	AKA	NA	poor knowledge n = 261(42%) adequate knowledge n = 559(58%)	Kruskal Wallis p = 0.399	Moderate quality

Abbreviations: AKA, Anticoagulation Knowledge Assessment questionnaire [30]; NA: Not Available; OAK, Oral Anticoagulation Knowledge test [31]; OR, odds ratio; r, Spearman correlation; TTR, time in the therapeutic range.

**Fig 2:**
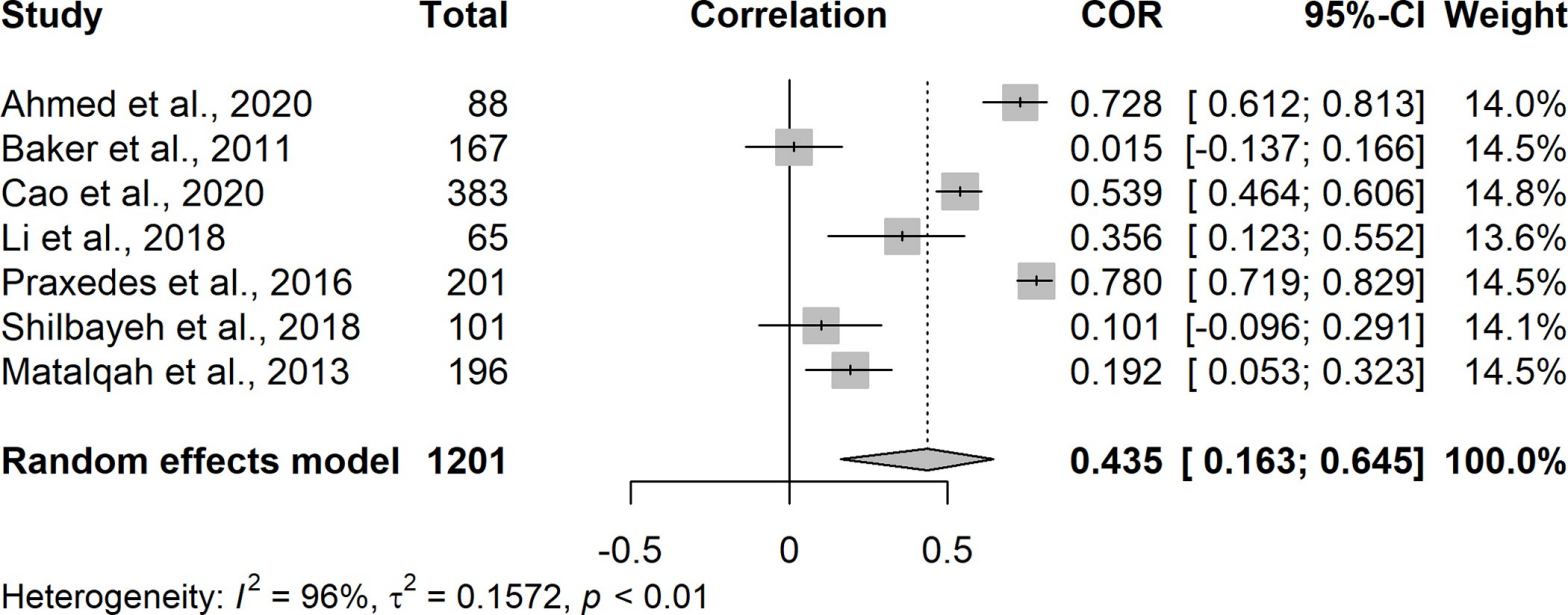
Forest plot of the correlation between the patient level of knowledge on warfarin therapy and oral anticoagulation control.

The meta-analysis was performed with seven [9,19–21,24,25,27] studies that analyzed the correlation between the patient level of knowledge on warfarin therapy with TTR employing the Spearman’s method. Pooled estimation indicated that patient level of knowledge on warfarin therapy was moderately associated with TTR (rs = 0.435; 95% CI = 0.163 to 0.645; I^2^ = 96%) (Fig 2).

**Fig 3:**
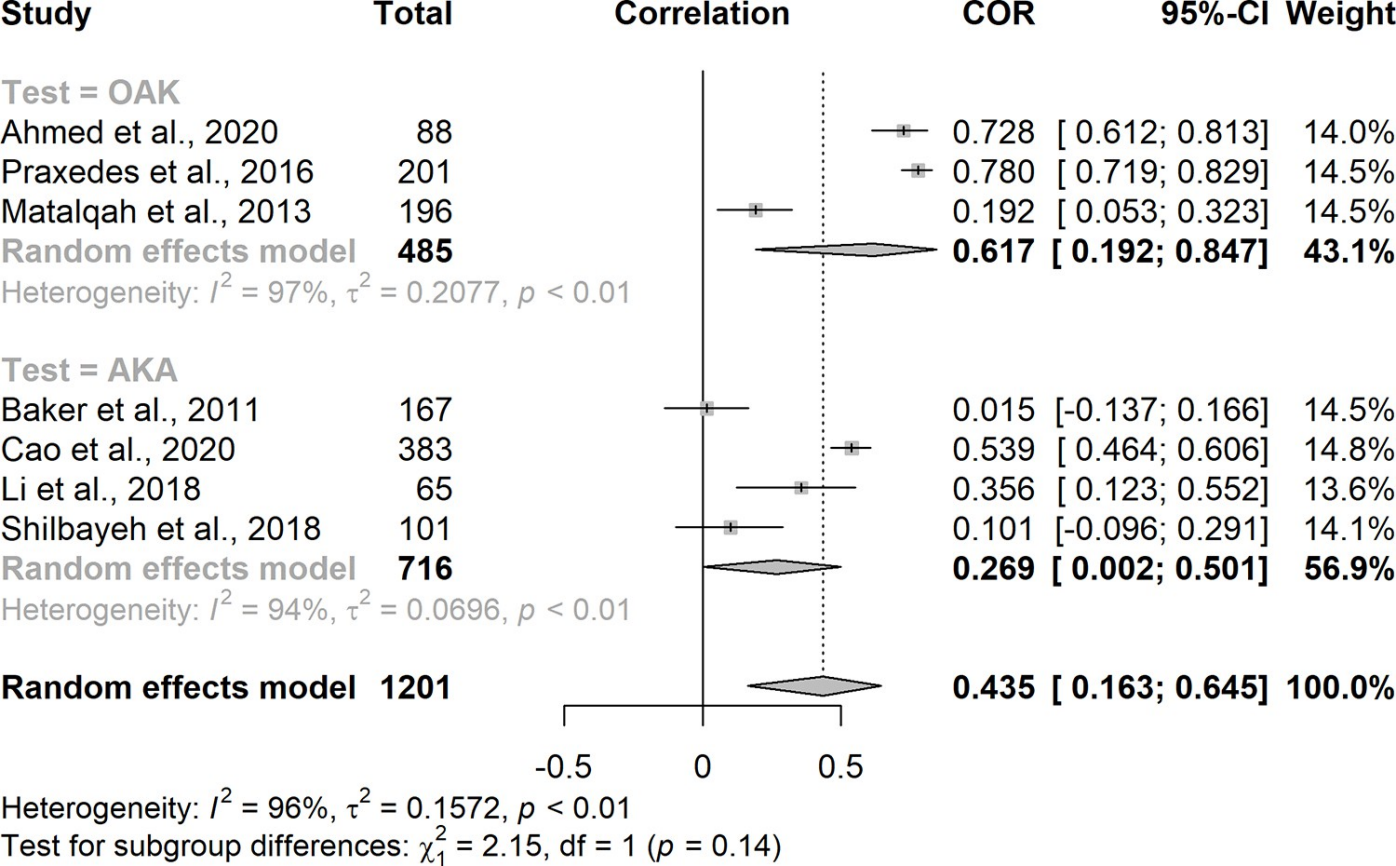
Forest plot of the correlation between the patient level of knowledge on warfarin therapy according to the methods of knowledge measurement.

Subgroup analysis indicated association between the patient level of knowledge on warfarin therapy and TTR in studies that employed the OAK test (rs = 0.617; 95% CI = 0.192 to 0.847; I^2^ = 97%) and the AKA (rs = 0.269; 95% CI = 0.002 to 0.501; I^2^ = 94%). However, the subgroup analysis showed no significant difference between them (p = 0.14) (Fig 3). No asymmetry was noted among the studies (p = 0.8806), suggesting no publication bias.

**Fig 4:**
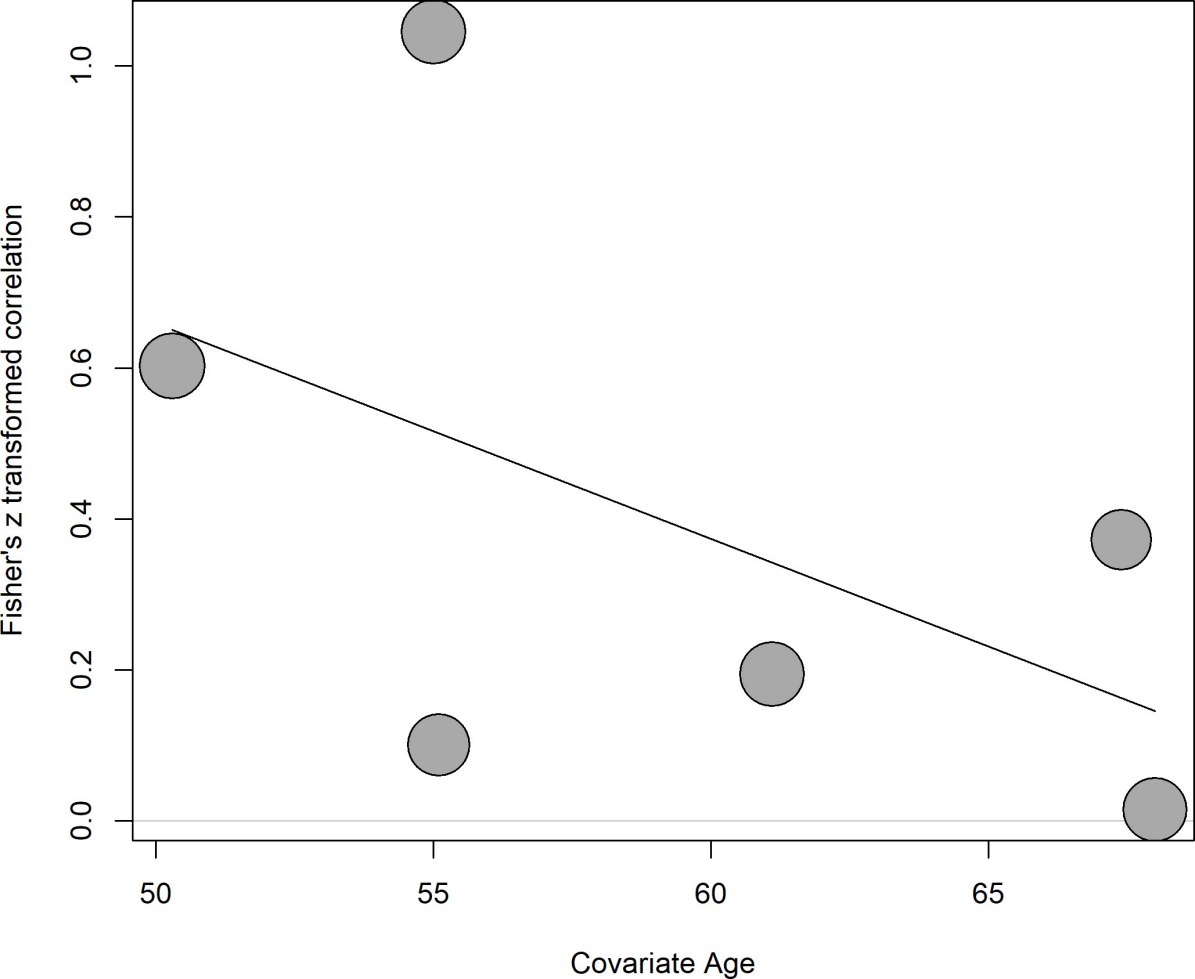
Bubble plot for meta-regression with age as continuous covariate.

The meta-regression showed a non-significant negative effect of age on the correlation (estimate = -0.028, 95% CI = -0.073 to 0.016, p = 0.207) (Fig 4). One study [19] was excluded from this analysis because of missing age. The residual heterogeneity decreased from Q = 151.40 to Q = 81.49. The percentage of total variability due to heterogeneity was I^2^ = 95.18%.

## Discussion

The present systematic review and meta-analysis gathered evidence about the evaluation of the relationship between the patient level of knowledge on warfarin therapy and the quality of warfarin therapy measured by TTR. Among the selected studies, there were different results regarding the relationship between the variables analyzed. Seven [9,19,21–25] studies indicated a significant association between the patient level of knowledge on warfarin therapy and TTR, whereas five [20,26–29] studies did not indicate statistical significance.

Such difference may be linked to the difficulty in associating these variables, since both can suffer independent interferences due to patients’ characteristics, such as age, sex, education, income, genetic polymorphisms, diet, concomitantly used medications, health literacy level. Besides, differences related to geographic regions, quality of health services and health education programs should also be considered [2,32–35]. Thus, the heterogeneity indicated by the metanalysis may be closely related to the difficulty of comparison among studies.

The selected studies stratified the patient level of knowledge on warfarin therapy, presenting levels ≥70–75% related to an adequate level of knowledge, depending on the instrument chose to measure patient knowledge. The overall average level of knowledge found in this study (60.4%). In line with this finding, studies have pointed out that the patients’ knowledge about warfarin therapy is still poor, even when educational interventions are provided [36,37]. This may be related to some factors, such as low health literacy and the complexity of the contents addressed by instruments, which often are not understood or memorized adequately [38]. The educational activities should use clear and accessible language according to the audience, highlighting the therapeutic objective (indication), the process of use (dosing regimen, route of administration, and duration of treatment), safety (adverse events, precautions contraindications and interactions), as well as conservation aspects for the drug [39].

Some selected studies pointed to a knowledge deficit predominantly in topics related to warfarin interactions with drugs, food and alcohol, to adverse events and dosing regimen. Incorrect answers identified by patient knowledge tests allow the identification of potential areas that can be explored by healthcare professionals to improve patient education and evaluate the effectiveness of the current educational activities [20,24]. Variables indicated as related to low patient knowledge were advanced age, low educational level, and less frequent INR monitoring [19,22]. Therefore, the provision of patient-centered interventions with structured and standardized methodology can greatly contribute to raising the patient level of knowledge on warfarin therapy [40].

The mean TTR identified in this study (57.8%) is below the value recommended by the literature, indicating that the sample studied is exposed to warfarin-related adverse events [41,42]. Patients with TTR >75% have been reported to present significantly lower bleeding and mortality rates compared to TTR <60% [43]. The TTR may suffer influence from the level of knowledge, as pointed out by our analyses. The use of validated tests capable of measuring the current level of knowledge can help stratifying patients and improve the planning and individualization of educational practices according to their needs. Thus, they tend to guarantee an increase in the level of knowledge and, consequently, in the TTR [9,32,33].

Another issue that stands out to the difficulty of associating the level of knowledge with TTR concerns the methodological differences of studies. We found variability in geographic regions, sample size, instruments used to measure the level of patient knowledge, as well as different stratifications for the level of knowledge considered appropriate for a population. Considering these variations, the meta-analysis was performed only with the seven studies [9,19–21,24,25,27] that indicated the Spearman’s rank correlation coefficient. With the exception of one study [22], all the others used validated instruments (AKA [30] and OAK [31]), in which, by subgroup analysis, there was no significant difference between them. The use of valid instruments is essential to generate reliable and comparable data [44]. In this sense, the analyses in this study become more comparable to each other.

The meta-regression showed a non-significant negative effect of age on the correlation researched. By analysis of the studies included in this review, there are conflicting results. Some studies pointed out a significant association [19,28], while others identified no association [9,25–27,29]. This demonstrates the need for further investigation of such a relationship, with studies designed specifically for this purpose.

In the present study, some limitations stand out. The small number of studies included indicate that potential eligible studies may have been left out of the initial selection, despite the fact that we followed the recommended methodology for systematic reviews in a rigorous manner. To meet the proposed aim and answer the research question, only observational studies were selected, and our results depended on their methodological quality. In the study protocol, the Web of Science database was initially planned to be included. However, the access to this database has been cancelled in our academic institution, possibly restricting our search. Moreover, we should consider the heterogeneity identified by the metanalysis, which may be related to differences inherent to the studies, such as sample size, geographic location, indication of warfarin use, among others. Furthermore, subgroup analysis was performed and the Begg-Mazumdar test did not indicate publication bias.

## Conclusion

To the best of our knowledge, this is the first systematic review and meta-analysis to gather evidence about the relationship between the patient level of knowledge on warfarin therapy and the quality of oral anticoagulation, expressed by TTR. From the analysis performed, a positive association was observed, in which the higher the level of knowledge, the higher the quality of warfarin therapy. This result can serve as a basis for healthcare professionals and public policy managers to reinforce the importance of structured and patient-centered educational interventions that effectively promote an increase in the level of knowledge of patients, thus ensuring better quality and safety of warfarin therapy.

## Supporting information

S1 Checklist PRISMA 2020 checklist. (DOCX)

S1 Appendix Terms and search strategies by database. (DOCX)
