# Supplementary material for: Assessment of the relationship between the level of patient knowledge on warfarin therapy and the quality of oral anticoagulation: A systematic review and meta-analysis
Source: PLoS One. 2023 Aug 10;18(8):e0289836. doi: 10.1371/journal.pone.0289836 (PMC10414645; doi:10.1371/journal.pone.0289836)
Supplement: S1 Appendix — (DOCX) [file pone.0289836.s002.docx]

| **Appendix 1**  **Terms and search strategies by database** | |
| --- | --- |
| **DATABASES** | **STRATEGY** |
| MEDLINE | #1 “Patient Medication Knowledge” [Mesh Terms] OR (Knowledge, Patient Medication) OR (Medication Knowledge, Patient) OR (Patient Drug Knowledge) OR (Drug Knowledge, Patient) OR (Knowledge, Patient Drug) OR “Patient Education as Topic” [Mesh Terms] OR ( Education, Patient) OR (Patient Education) OR (Education of Patients) OR “Health Education” [Mesh Terms] OR (Education, Health) OR (Community Health Education) OR (Education, Community Health) OR (Health Education, Community)  #2 Warfarin [Mesh Terms] OR ( 4-Hydroxy-3-(3-oxo-1-phenylbutyl)-2H-1-benzopyran-2-one) OR (Apo-Warfarin) OR (Aldocumar) OR (Gen-Warfarin) OR (Warfant) OR (Coumadin) OR (Marevan) OR (Warfarin Potassium) OR (Potassium, Warfarin) OR (Warfarin Sodium) OR (Sodium, Warfarin) OR (Coumadine) OR (Tedicumar)  #3 #1 AND #2 |
| EMBASE | 'patient education'/ exp OR 'patient education'/ syn OR 'health education'/exp OR 'health education'/syn AND ‘warfarin'/ |
| LILACS | ("Conhecimento do Paciente sobre a Medicação" OR "Patient Medication Knowledge" OR "Conocimiento de la Medicación por el Paciente" OR "Connaissance des patients sur la medication" OR "Educação de Pacientes como Assunto" OR "Patient Education as Topic" OR "Educación del Paciente como Asunto" OR "Éducation du patient comme sujet" OR "Educação de Pacientes" OR "Educação do Paciente" OR "Educação em Saúde" OR "Health Education" OR "Educación en Salud" OR "Éducation pour la santé" OR "Patient Education") AND (varfarina OR warfarin OR warfarina OR warfarine) AND ( db:("LILACS" OR "IBECS" OR "BDENF")) |
| Scopus | ("Patient Medication Knowledge" OR "Patient Education as Topic" OR "Health Education" OR "Patient Education") AND (Warfarin) |
| WEB OF SCIENCE | ("Patient Medication Knowledge" OR "Patient Education as Topic" OR "Health Education" OR "Patient Education") AND (Warfarin) |
